# A case of scrub typhus with meningitis as the onset: Case report and literature review

**DOI:** 10.1097/MD.0000000000038613

**Published:** 2024-06-21

**Authors:** Bing-Can Zhang, Zi-Bin Yang, Ren-Li Liao, Zhi-Qiang Ma, Qiu-Juan Zhang, Qian-Kun He, Xin-Ya Duan, Ming-Wei Liu

**Affiliations:** a Department of Orthopedics, People’s Hospital of Dali Bai Autonomous Prefecture, Dali, Yunnan, China; b Department of Clinical Laboratory, People’s Hospital of Dali Bai Autonomous Prefecture, Dali, Yunnan, China; c Department of Emergency, The First Affiliated Hospital of Kunming Medical University, Kunming, Yunnan, China; d Department of Neurology, The First Affiliated Hospital of Guizhou University of Traditional Chinese Medicine, Guiyang, China; e Department of Tuberculosis Diseases, Third People’s Hospital of Kunming City, Kunming, China; f Department of Emergency, The People's Hospital of Dali Bai Autonomous Prefecture, Dali, Yunnan, China.

**Keywords:** case report, central nervous system infection, diagnosis, scrub typhus, treatment

## Abstract

**Rationale:**

Scrub typhus is a naturally occurring acute febrile disease caused by *Orientia tsutsugamushi*. Although it can cause multiple organ dysfunction, central nervous system infections are uncommon.

**Patient concerns:**

A 17-year-old male presented with a 5-day history of fever and headaches. The MRI of the head revealed thickness and enhancement of the left temporal lobe and tentorium cerebelli, indicating potential inflammation.

**Diagnoses:**

The patient was diagnosed with a central nervous system infection.

**Interventions:**

Ceftriaxone and acyclovir were administered intravenously to treat the infection, reduce fever, restore acid-base balance, and manage electrolyte disorders.

**Outcomes:**

Despite receiving ceftriaxone and acyclovir as infection therapy, there was no improvement. Additional multipathogen metagenomic testing indicated the presence of *O tsutsugamushi* infection, and an eschar was identified in the left axilla. The diagnosis was changed to scrub typhus with meningitis and the therapy was modified to intravenous doxycycline. Following a 2-day therapy, the body temperature normalized, and the fever subsided.

**Conclusions:**

The patient was diagnosed with scrub typhus accompanied by meningitis, and doxycycline treatment was effective.

**Lession:**

Rarely reported cases of scrub typhus with meningitis and the lack of identifiable symptoms increase the chance of misdiagnosis or oversight. Patients with central nervous system infections presenting with fever and headache unresponsive to conventional antibacterial and antiviral treatment should be considered for scrub typhus with meningitis. Prompt multipathogen metagenomic testing is recommended to confirm the diagnosis and modify the treatment accordingly.

## 1. Introduction

Scrub typhus, also known as bush typhus, is an acute infectious disease caused by *Orientia tsutsugamushi*. It is characterized by eschar at the bite site, high fever, lymph node enlargement, rash, and multisystem involvement, and is particularly responsive to doxycycline. Currently, about 1 billion people globally are susceptible to scrub typhus, with approximately 1 million new cases reported annually. The majority of cases occur in “*tsutsugamushi* triangle,” mainly in Southeast Asia, northern Australia, and the Asia-Pacific area.^[1]^ Scrub typhus has historically been more widespread in the southern region of China, while its cases have been on the rise in different areas owing to agricultural activities in recent years.^[2]^ Owing to its variable clinical manifestations and patient-specific severity, scrub typhus is susceptible to misdiagnosis and failure to diagnose. The mortality rate remains 1.4% despite treatment,^[3]^ whereas it increases to 6% in untreated patients.^[4]^ Concurrent infection of the central nervous system may result in a mortality rate of up to 14%.^[5]^ Tsutsugamushi disease can occur year-round, with the southern focus mainly prevalent in summer, and the Guangdong region south of 25° N latitude experiences outbreaks throughout the year. In Yunnan, tsutsugamushi disease mostly occurs in summer and autumn, from May to October, with a peak from June to July. This was related to the spread of ground chigger mites caused by concentrated rainfall.

Studies have shown that scrub typhus often leads to multisystem dysfunction in the second week of onset, manifested as myocardial injury, interstitial pneumonia, meningitis or meningoencephalitis, pancreatitis, abnormal liver function, acute kidney injury, and optic neuritis.^[6]^ It was uncommon for this patient to manifest symptoms and signs of central nervous system infection from the beginning. This case report aims to guide clinicians in the diagnosis and treatment of scrub typhus with central nervous system involvement as the first symptom. The goal is to minimize misdiagnosis, ensure prompt diagnosis and treatment of these patients, and enhance their prognoses.

## 2. Case report

### 2.1. Ethics approval and consent to participate

Informed written consent was obtained from the patient for the publication of this case report and accompanying images.

This study was reviewed and approved by the local ethics committee of the First Affiliated Hospital of Kunming Medical University. The procedures were in accordance with the Helsinki Declaration of 1975, as revised in 2000.

### 2.2. Medical history

A 17-year-old male patient presented with a diffuse, persistent, and whole-head headache that lasted for 5 days without any apparent etiology. Additionally, the patient experienced general weakness and multiple episodes of vomiting with a body temperature of 41°C. He was diagnosed with an “upper respiratory tract infection” and was administered an intravenous infusion at a local hospital; however, his condition did not improve. Therefore, he was admitted to the emergency department of the First Affiliated Hospital of Kunming Medical University for head MRI and contrast enhancement. The diagnosis of “possible central nervous system infection” led to his admission to the neurology department of the same hospital for treatment.

### 2.3. Past medical history

The patient had undergone axillary osmidrosis surgery half a year ago. He denied having hypertension, diabetes, cardiovascular disease, tuberculosis, influenza, typhoid fever, or any other infectious diseases in his medical history. His medical history did not include any documented surgical procedures, blood transfusions, or dietary or drug allergies. The vaccination history was unknown. The patient reported no family history of infectious diseases.

### 2.4. Physical examination

The patient had a body temperature of 38.8°C, pulse rate of 128 beats/min, respiratory rate of 21 breaths/min, and blood pressure of 128/74 mm Hg. His general condition was good with no enlargement of the superficial lymph nodes throughout the body. He displayed consciousness, large and round bilateral pupils with a diameter of 4 mm, responsive to light reflex, normal elevation of bilateral soft palate, centralized uvula, responsive pharynx reflex, bilateral shrug, inability to turn the neck, absence of sternocleidomastoid muscle atrophy, centralized tongue extension, no signs of tongue muscle atrophy or muscle fasciculation, neck stiffness, 4-finger distance between the chin and sternum, and positive meningeal irritation sign. The patient exhibited a Kernig sign (−), normal muscle tone in all 4 limbs, grade 5 muscle strength in all 4 limbs, symmetrical tendon reflex (+), bilateral Babinski (−), intact needle-pricking pain sensation, steady and accurate bilateral finger-nose test and heel-knee-shin test, Romberg sign (−), mRS score of 4, and no apparent anxiety or depression.

### 2.5. Laboratory data

The results of a routine blood test underwent on August 29, 2019 were as follows: White Blood Cell count (WBC), 8.16 × 10^9^/L; Neutrophils (N), 81.8%; Lymphocytes (L), 14.1%; Eosinophils: 0.00%, Red Blood Cell count (RBC), 6.8 × 10^12^/L; Hemoglobin, 167g/L; Absolute Neutrophil count (ANC), 6.8 × 10^9/^L; Platelet count, 31 × 10^9^/L; Results of biochemical blood tests demonstrated that Sodium, 125.82 mmol/L; Chlorine 95.14 mmol/L, Alanine aminotransferase (ALT), 222.00IU/L; Aspartate aminotransferase (AST), 327.00IU/L; AST/ALT ratio, 1.47; Direct bilirubin (DBIL), 13.7 µmol/L; Creatinine (Cre), 115.90 µmol/L; Urea nitrogen (BUN), 7.8 µmol/L; Sodium, 123.12 mmol/L; Chlorine, 94.65 mmol/L, Prothrombin time (PT), 16.2 seconds; International normalized ratio (INR), 1.32; PT ratio, 1.25%; International sensitivity index (ISI), 1.26; Activated partial thromboplastin time (aPTT) 55.4 seconds. The results of the infection-related protein tests indicated the following values: procalcitonin, 10.21 ng/mL ↑; High-sensitivity C-reactive protein, 174.00 mg/L ↑. Cerebrospinal fluid analysis revealed the following: colorless and transparent appearance; Pan test negative (−); RBC, 41 × 10^9^/L; WBC, 9 × 10^12^/L. The biochemical analysis of cerebrospinal fluid on August 30, 2019, revealed the following results: Chlorine, 118.80 mmol/L ↓; trace total protein, 0.570 g/L ↑; trace Albumin 259.3 mg/L↑. Myocardial enzyme, urine analysis, systemic lupus erythematosus, rheumatoid arthritis-specific antibodies, anti-cardiolipin antibodies, and anti-neutrophil cytoplasmic antibodies tested negative. Tests for novel coronavirus and influenza nucleic acids were negative.

### 2.6. Medical imagology

**Figure 1.**
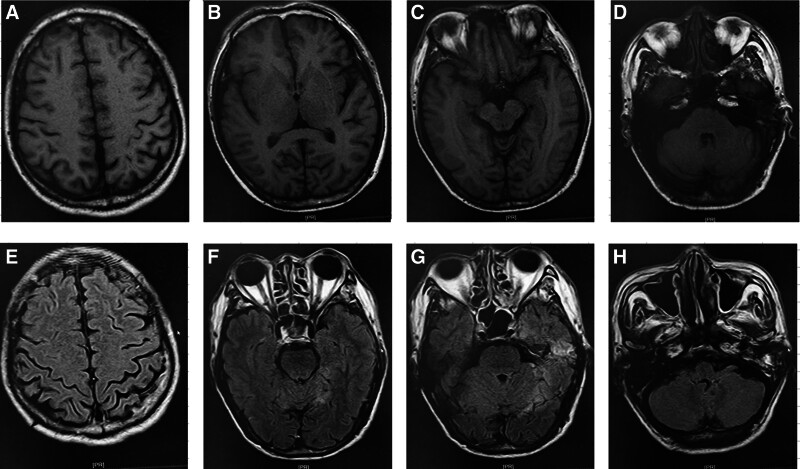
The alterations in the patient head MRI. (A–E) the alternations of the patient nuclear magnetic resonance (MRI) upon admission; (H–J) the alternations of the patient head Computer tomography (CT) 6 months after discharge.

A head MRI performed on August 29, 2019, illustrated thickening and enhancement of the dura mater and cerebellar tentorium adjacent to the left temporal lobe, indicating possible inflammation (Fig. 1). Mild enhancement was observed in the leptomeninges of the left temporal parietal lobe, indicating leptomeningitis (Fig. 1). A few white matter demyelination alterations were observed in the bilateral frontal parietal lobe cortices (Fig. 1).

### 2.7. Diagnosis and treatment

**Figure 2.**
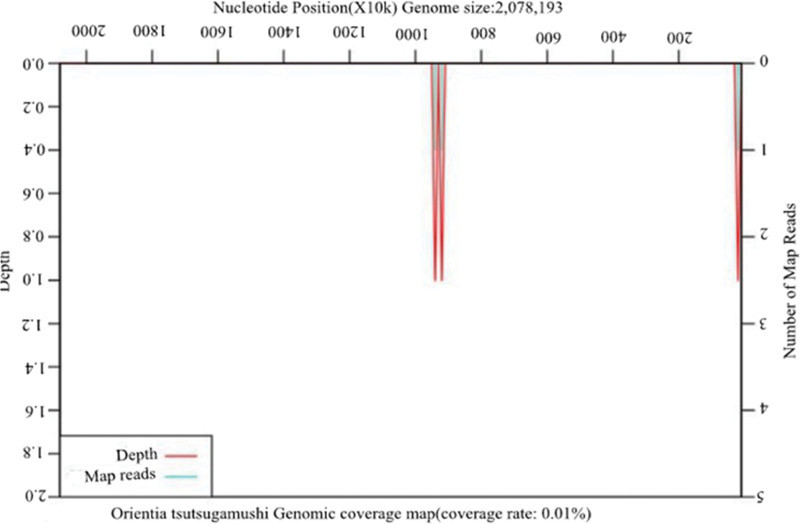
Genome coverage map of *Orientia tsutsugamushi.*

**Figure 3.**
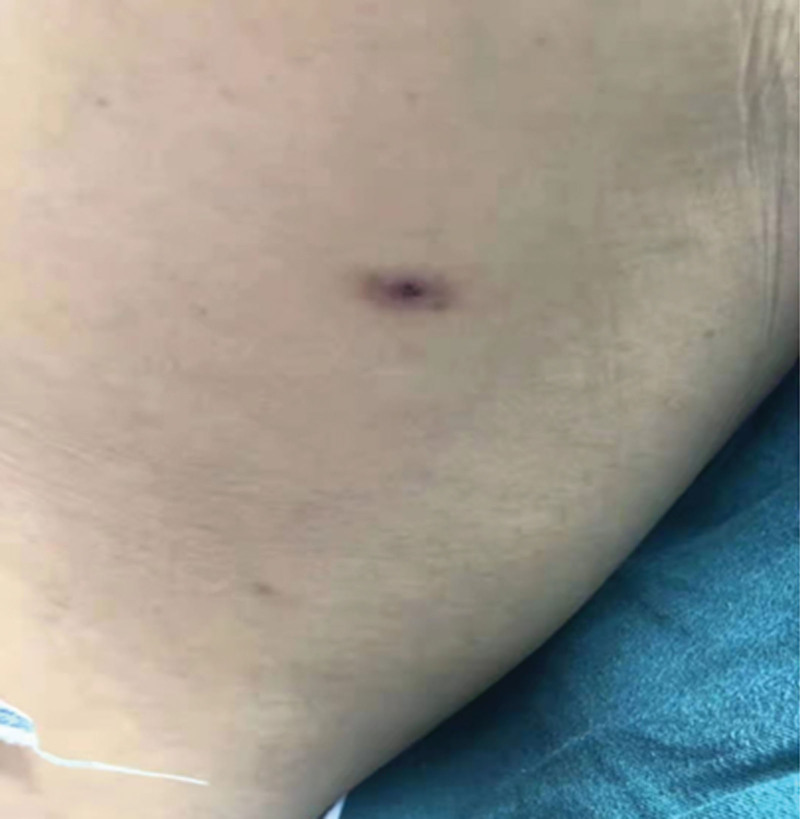
Eschars were found in the left axilla.

The patient was diagnosed with central nervous system infection based on his medical history, symptoms, signs, and laboratory data. The patient received an infusion of ceftriaxone 2 g/d, twice a day and acyclovir (0.5 g) 3 times a day for 3 days as anti-infective therapy, but did not show any improvement. Additional multipathogen metagenomic analysis identified *O tsutsugamushi* (Table 1 and Fig. 2), whereas no other microorganisms such as bacteria, fungi, DNA/RNA viruses, or parasites were found (Table 1). Furthermore, an eschar was observed in the left axilla (Fig. 3), leading to a revised diagnosis of scrub typhus with meningitis. Ceftriaxone and acyclovir were discontinued, and doxycycline 0.1 g/d was administered intravenously twice daily. The patient body temperature returned to normal, and no fever developed after 2 days of treatment. After receiving another 2-day treatment, the patient wanted to be discharged for continuing treatment at home. The patient was advised to maintain the regimen of taking doxycycline 0.1 g/d twice daily and to have a follow-up appointment after 1 week of therapy.

**Table 1:** Results of multiple pathogen metagenomic testing.

Pathogenic microorganism metagenomic detection report												
Tel: 4001-111-120 Website: www.kingmed.com.cn												

1. List of special pathogens (mycobacterium, mycoplasma, chlamydia, rickettsia, spirochetes, etc.)												
Category							Composite group/species					
Types	Name		Sequence number		Relative abundance		Name			Sequence number		Coverage
G-	*Orientia*		4		0.22%		*Orientia tsutsugamushi*			4		0.01%

2. List of bacteria												
Category							Composite group/species					
Types	Name		Sequence number		Relative abundance		Name			Sequence number		Coverage
Not detected												

3. List of fungi												
Category							Composite group/species					
Types	Name		Sequence number		Relative abundance		Name			Sequence number		Coverage
Not detected												

4. List of DNA/RNA viruses												
Category							Composite group/species					
Types	Name		Sequence number		Relative abundance		Name			Sequence number		Coverage
Not detected												

5. List of parasites												
Name						Sequence number			Relative abundance			
Not detected												

6. List of suspected colonization and/or background microorganisms												
Category								Composite group/species				
Types		Name		Sequence number				Name			Sequence number	
G-		*Moraxella*		18				*Moraxella osloensis*			18	
G+		*Staphylococcus*		11				*Staphylococcus epidermidis*			4	

7. List of resistance genes												
Resistant gene		Corresponding species		Sequence number				Coverage			Corresponding resistant antimicrobial drugs	
Not detected												

### 2.8. Follow-up after treatment

Two weeks after discharge for treatment, the patient exhibited no discomfort, and no anomalies in liver or kidney function were detected during follow-up.

## 3. Discussion

Scrub typhus is an azoonotic disease caused by *O tsutsugamushi*, and is mainly transmitted by chiggerbites; subsequently, *O tsutsugamushi* gains access to the body via macrophage phagocytosis. During the acidification process, *O tsutsugamushi* escapes from the phagosome, replicates via binary fission in the cytoplasm, and ultimately escapes the phagocyte by budding and acquiring a part of the host cell membrane.^[7]^ This process leads to further infection of nearby cells, as well as spreading to various organs via blood and lymph circulation, resulting in organ-specific inflammatory reactions, tissue damage, and multiple organ failure. Murhekarmv et al^[8]^ showed that the average time for central nervous system symptoms to appear in patients with scrub typhus was 6 days after fever. This was primarily attributed to *O tsutsugamushi* proliferation in the meninges, leading to vasculitis and inflammation around capillaries, small arteries, and arterioles. Upon microscopic examination, histiocytes, L, and plasma cells had infiltrated the meningeal and vascular peripheral spaces. The extent of infiltration was correlated with the intensity of the inflammatory response. Common serious complications in the past usually occurred in the second week after onset,^[9]^ including severe pneumonia, acute renal insufficiency, meningitis, encephalitis, gastrointestinal hemorrhage, septic shock, multiple organ dysfunction syndrome (MODS), and mortality. The first symptom in this case was central nervous system infection, which is relatively rare.

Scrub typhus infection follows a clear seasonal pattern, with the highest occurrence in July-August and October-November in China.^[10]^ This is linked to the rise in reproductive density of chiggers and mice during these months. It is prevalent in alpine grasslands, tropical forests, sandy beaches, and several other regions, and is distributed over much of southern China. The incidence rate has recently increased. Rodents are common hosts for *O tsutsugamushi*, with Apodemus agrarius serving as the predominant species in Yunnan.^[11]^ Chiggers act as vectors of transmission to humans. It is prevalent across all age groups but tends to be more frequent in young adults and children, with a somewhat higher occurrence in men than females.^[12]^ Farmers and fieldworkers (lumberjacks, road builders, geological explorers, and avid anglers) are more likely to be affected by increased exposure to chigger bites. Yunnan Province is a significant endemic region for scrub typhus owing to its specific geographical position and climate. Most cases are seen in adults aged between 40 and 65 years.^[12,13]^ This patient was in Yunnan, China, where scrub typhus is widespread, and the onset of symptoms occurs in August. The patient also had a history of entering the bush.

The symptoms of scrub typhus are often atypical and mostly manifest as fever, respiratory symptoms, central nervous system infection, digestive tract symptoms, or myocarditis,^[12]^ which can easily be misdiagnosed as unexplained fever, pneumonia, upper respiratory tract infection, and encephalitis. This can lead to delayed diagnosis and progression to multiple organ failure. Upon admission to the hospital, the patient was incorrectly diagnosed with a central nervous system infection based on the symptoms of fever, headache, and head MRI. Treatment with ceftriaxone and acyclovir was unsuccessful. Additional multipathogen metagenomic analyses identified *O tsutsugamushi*. The primary causes of misdiagnosis included insufficient clinical experience, lack of knowledge about the disease, and lack of attentiveness; inadequate gathering of clinical information and incomplete physical examination, particularly oversight of common eschar sites such as the inguinal region and perineum; and being deceived by certain prominent symptoms and signs. Symptoms, such as chills, high fever, low white blood cell count, and splenomegaly, are often misdiagnosed as typhoid fever. Misdiagnosis can be reduced by conducting thorough medical history assessments, rigorous physical examinations, and epidemiological data analyses. Skin and mucosal eschar or ulcers are the most typical features in diagnosing this disease and are seen in over 90% of patients.^[14]^ Scabs are mainly distributed in the groin, armpits, trunk, perineum, limbs, and other parts and are not easily detected. Therefore, eschar may be missed in individuals with dark skin. Following unsuccessful therapy with ceftriaxone and acyclovir, the patient underwent a thorough examination, which revealed an eschar located near the left axilla, confirming the diagnosis.

Currently, laboratory and clinical diagnoses are the 2 most common methods of scrub typhus. Diagnostic criteria are as following^[15]^: History of field activities for the last 3 weeks; Fever; Eschar or ulcer; Hepatosplenomegaly or lymph node enlargement; Weil-Felix test ≥ 1:160; When all 3 criteria mentioned above are fulfilled simultaneously, a diagnosis of scrub typhus can be made, while it is necessary to exclude typhoid fever, epidemic hemorrhagic fever and other diseases. A high degree of clinical suspicion based on season and region is very important. The patient had a history of field activities, fever, eschar, and epidemics. After ruling out typhoid fever and epidemic hemorrhagic fever, a diagnosis of scrub typhus was confirmed.

Molecular biology techniques include polymerase chain reaction (PCR), recombinase polymerase amplification (RPA), and metagenomic next-generation sequencing (mNGS). Among these, mNGS stands out for its rapid detection time (<24 hours), exceptional sensitivity, and specificity.^[16]^ This method is valuable for early and accurate diagnosis of scrub typhus and other uncommon microbial infections. In this case, given the patient symptoms consistent with a central nervous system infection, the head MRI suggested meningitis, and the unsuccessful treatment for meningitis, mNGS was promptly conducted to identify *O tsutsugamushi* and no bacteria, fungus, DNA/RNA viruses, or parasites were discovered. Therefore, the patient was diagnosed as having scrub typhus.

The mortality rate of severe scrub typhus was 24.1%.^[12]^ Prompt and efficient diagnosis and treatment may lower the mortality rate of scrub typhus.^[12]^ *O tsutsugamushi* is a gram-negative intracellular bacterium that exhibits inherent resistance to many popular antibiotics, such as β-lactams, fluoroquinolones, and aminoglycosides. However, it is susceptible to lipophilic tetracycline drugs that can enter the host cell membrane. Hence, it can be used to treat scrub typhus quickly and effectively, with a more favorable outcome when treated early. It can effectively shorten the course of the disease and reduce mortality.^[17]^ Doxycycline is currently the primary therapy for scrub typhus. Patients with mild scrub typhus were administered oral doxycycline (0.1 g twice a day for a week. Severely ill patients should receive intravenous doxycycline, azithromycin alone, or a combination of oral doxycycline.^[18]^

### 3.1. Strengths and limitations of this study

Strengths: Patients with fever who do not have eschar or are unable to locate eschar should undergo early multiple pathogen metagenomic testing for precise diagnosis. Doxycycline is effective in treating confirmed cases of scrub typhus.

Limitations: Scrub typhus accompanied by meningitis is seldom reported, with symptoms that are nonspecific, increasing the likelihood of missed diagnosis and misdiagnosis. Furthermore, the pathogenesis of meningitis caused by scrub typhus is not well understood and requires further investigation.

## 4. Conclusion

CNS involvement is common in Srub.^[19]^ However, atypical symptoms increase the likelihood of oversight and misdiagnoses. Patients with a central nervous system infection, fever, and headache unresponsive to bacterial and viral treatments should be considered for scrub typhus with meningitis. A thorough examination is needed to check for eschar on the whole body, and multiple pathogen metagenomic testing should be performed promptly to confirm the diagnosis and promptly adapt the therapy. The case highlights the need for a good history and extensive physical examination, which could have resulted in the diagnosis in this case even before the results of expansive investigations (especially for young inexperienced physicians). Doxycycline should be administered promptly to patients diagnosed with scrub typhus causing meningitis.

## Author contributions

**Conceptualization:** Zi-Bin Yang, Ming-Wei Liu.

**Data curation:** Bing-Can Zhang, Zi-Bin Yang, Ren-Li Liao, Zhi-Qiang Ma, Qiu-Juan Zhang, Ming-Wei Liu.

**Formal analysis:** Bing-Can Zhang, Xin-Ya Duan.

**Funding acquisition:** Zhi-Qiang Ma, Qiu-Juan Zhang, Qian-Kun Qian, Xin-Ya Duan, Ming-Wei Liu.

**Investigation:** Bing-Can Zhang, Zi-Bin Yang, Ren-Li Liao.

**Methodology:** Zhi-Qiang Ma, Qiu-Juan Zhang, Qian-Kun Qian.

**Project administration:** Zi-Bin Yang, Ming-Wei Liu.

**Resources:** Zi-Bin Yang, Ren-Li Liao, Zhi-Qiang Ma, Qiu-Juan Zhang, Xin-Ya Duan.

**Software:** Zi-Bin Yang, Ren-Li Liao, Qian-Kun Qian, Ming-Wei Liu.

**Supervision:** Bing-Can Zhang, Zi-Bin Yang, Zhi-Qiang Ma, Qiu-Juan Zhang, Qian-Kun Qian, Xin-Ya Duan.

**Validation:** Zhi-Qiang Ma, Qiu-Juan Zhang, Qian-Kun Qian, Ming-Wei Liu.

**Visualization:** Bing-Can Zhang, Ren-Li Liao, Xin-Ya Duan.

**Writing – review & editing:** Zhi-Qiang Ma, Ming-Wei Liu.

**Writing – original draft:** Ming-Wei Liu.
